# Transferable neuronal mini-cultures to accelerate screening in primary and induced pluripotent stem cell-derived neurons

**DOI:** 10.1038/srep08353

**Published:** 2015-02-10

**Authors:** Mark Niedringhaus, Raluca Dumitru, Angela M. Mabb, Yuli Wang, Benjamin D. Philpot, Nancy L. Allbritton, Anne Marion Taylor

**Affiliations:** 1 UNC/NCSU Joint Department of Biomedical Engineering; 2 UNC Department of Cell Biology and Physiology; 3 UNC Department of Chemistry; 4 UNC Neuroscience Center; 5 Carolina Institute for Developmental Disabilities; 6 UNC Human Pluripotent Stem Cell Core; 7 UNC Department of Genetics

## Abstract

The effort and cost of obtaining neurons for large-scale screens has limited drug discovery in neuroscience. To overcome these obstacles, we fabricated arrays of releasable polystyrene micro-rafts to generate thousands of uniform, mobile neuron mini-cultures. These mini-cultures sustain synaptically-active neurons which can be easily transferred, thus increasing screening throughput by >30-fold. Compared to conventional methods, micro-raft cultures exhibited significantly improved neuronal viability and sample-to-sample consistency. We validated the screening utility of these mini-cultures for both mouse neurons and human induced pluripotent stem cell-derived neurons by successfully detecting disease-related defects in synaptic transmission and identifying candidate small molecule therapeutics. This affordable high-throughput approach has the potential to transform drug discovery in neuroscience.

Large-scale *in vitro* screening techniques have helped to advance basic research and identify numerous drugs and drug candidates for treating human diseases, particularly in the field of oncology^1^,^2^,^3^,^4^,^5^,^6^,^7^. Unfortunately, current methodologies are optimized for dividing cells that can be expanded to generate large sample sizes and not for post-mitotic cells such as neurons. The high cell densities required for proper functional network development have traditionally required the use of large numbers of neurons–up to 70,000 neurons/screen in a 384-well plate^8^. Furthermore, well-plates with smaller well volumes are not compatible with long-term cultures due to evaporative effects^9^. Neurons from transgenic disease-model animals or differentiated from patient-derived induced pluripotent stem cells (iPSCs) are available in limited quantities; obtaining sufficient neurons for large-scale screens has been impractical. Thus, despite available sources of neurons that model human neurological disease, large-scale screening studies in neurons remain rare and costly. With the goal of improving the pace of neuronal drug discovery efforts, here we provide an affordable approach to enable high-throughput screening in either rodent neurons or in human iPSC-neurons.

## Results

### Plating, release, and transfer of micro-rafts

We wanted to limit the number of neurons for each culture well, as this would allow many more neuronal culture wells to be generated and screened from an initial pool of neurons. At the same time, we sought to culture neurons at a sufficiently high density to promote synaptic connectivity and neuronal maturation. It is not feasible to use higher density well-plates, such as the 1,536-well format, because of detrimental evaporative losses due to the high surface area-to-volume ratio present in these minute well volumes. Our approach to increase samples size with limited material was to use microfabricated raft arrays^10^ (movie S1). We hypothesized that this technology would create cultures with sufficient neuronal density for network maturation as well as minimal, yet effective, numbers of neurons for compound screening.

**Figure 1:**
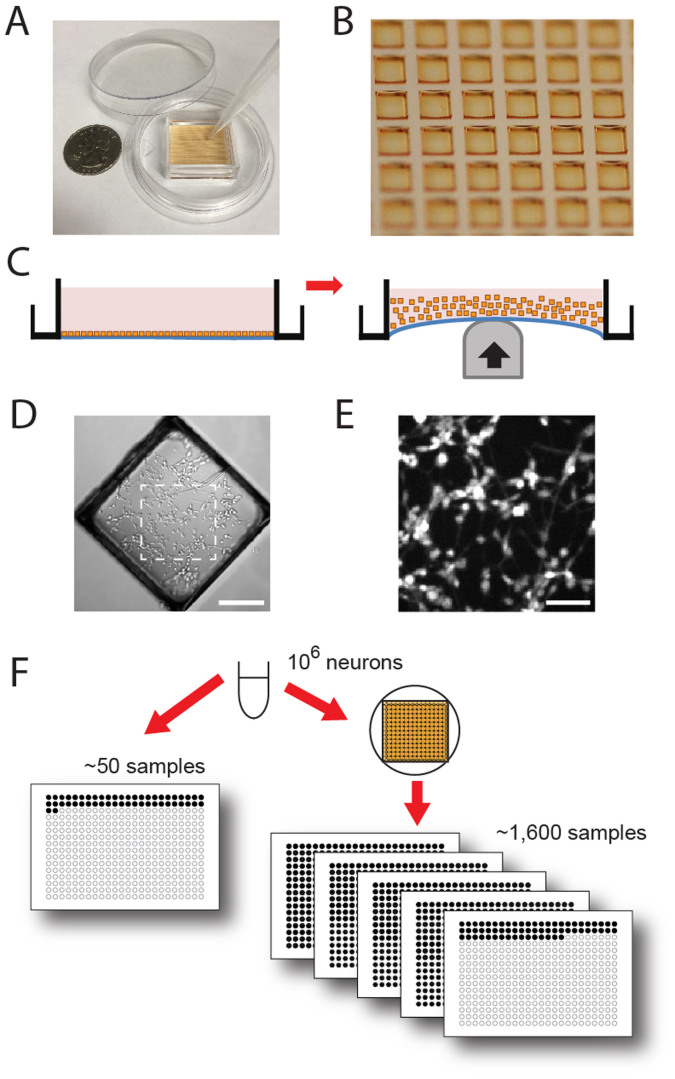
Each micro-raft array can generate thousands of separate and mobile neuron mini-cultures, including neuron cultures differentiated from human embryonic stem cells (ESCs). (A) Photograph of the micro-raft array and housing. Each array has 1,600 releasable rafts embedded in a flexible PDMS matrix. (B) A higher magnification image of microraft array (each raft is 500 μm x 500 μm) within the PDMS matrix, prior to release. (C) Cartoon depiction of raft release using a blunt probe to deflect the PDMS matrix, releasing the rigid polystyrene raft mini-cultures into the media. (D) DIC image of a representative raft micro-culture containing human ESC-neurons, released from the array at day 1 and grown on a released raft for an additional 18 days. Scale bar, 150 μm. (E) zoom of dashed box in *(D)* stained with live cell dye, Celltracker AM. Scale bar, 50 μm. (F) Micro-raft arrays increase screening potential >30-fold compared to using well-plates directly.

Each raft array consists of 1,600 releasable, polystyrene, ferromagnetic rafts (500 μm x 500 μm x 100 μm) cast in a flexible poly(dimethylsiloxane) (PDMS) matrix and housed within a fluid reservoir suitable for holding cell culture media (Fig. 1A–B). Rather than plating each surface individually as done in well-plates, approximately one million dissociated neurons are plated onto a single array (initial plating density: 1,600 neurons/mm^2^ = 400 neurons/raft), and after one to two days the rafts are released, *en masse*, into cell culture media reservoirs where they are cultured together for days or weeks to attain proper maturity with negligible evaporative losses (Fig. 1C-E). Rafts are then sorted as needed into high content well plates for compound screening via pipette and used for treatment or staining. Alternatively, rafts can be transferred via a magnetic wand^11^. A magnetic plate placed under the well plate quickly directs the magnetic raft to the bottom of the well. Importantly, once rafts are located to the floor of the well, they stay in place during normal transport or automated stage movements. The localization of these rafts at the bottom of the well also allows for imaging via high resolution microscopy and makes the rafts compatible with auto-focus functions. To facilitate fluid exchanges and treatment protocols, rafts are held in place on the well floor with a plate magnet. The slight concavity of the raft (Fig. 1D) and the surface tension of the media surrounding the raft protect neurons during release and transfer, without compromising the high-content imaging required for most screening strategies. Unlike contact spotting, which has been used to generate small-sized cultures for non-neuronal cell types^12^,^13^,^14^, the mobile raft mini-cultures can be independently screened in parallel. This method increases screening potential >30-fold over traditional well-plate cultures, while remaining compatible with well-plate-based high-throughput drug screening equipment (Fig. 1F).

### Mobile mini-cultures of human stem cell-neurons

**Figure 2:**
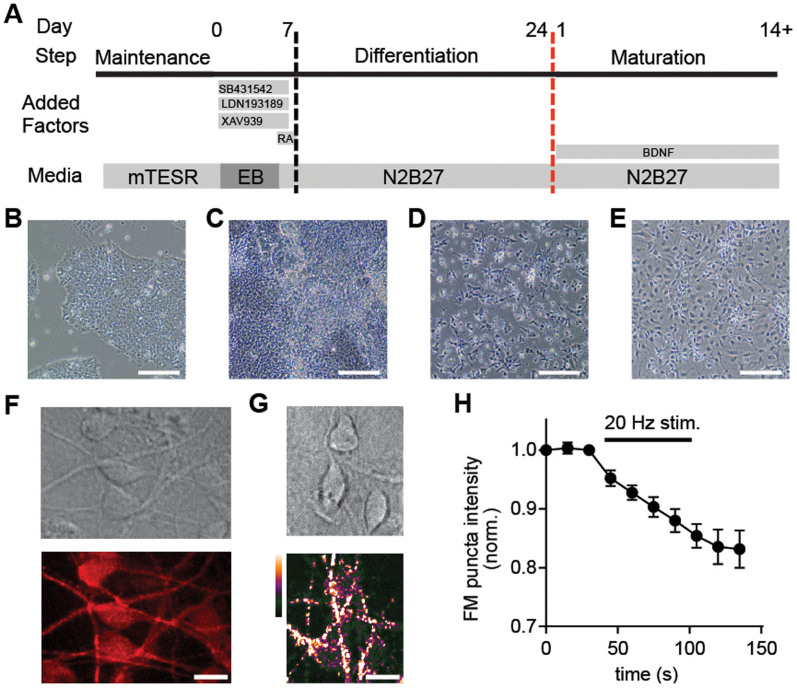
Human ESC cells differentiate into glutamatergic neurons. (A) Schematic of the stem cell neuron differentiation protocol. Days are numbered 0–24 for neuronal differentiation into neural progenitor cells, and then renumbered following final plating during neuronal maturation (see methods for details). Solution was replaced every day with the appropriate media plus factors listed above. Vertical, dashed lines indicate cellular plating; the red line indicates final plating into raft arrays or well plates. Representative micrographs of cells during (B) maintenance, (C) differentiation (day 10), and (D, E) maturation (day 1 and 10, respectively). Scale bars, 100 μm. (F) Mature, stem cell-neurons were MAP2-positive and (G) exhibited robust VGLUT1 expression on released rafts. (H) To test for functional activity-dependent synaptic vesicle release in stem cell-neurons, presynaptic terminals were loaded with FM dye (5–95) with KCl and unloaded with electrical stimulation (see methods for details), showing activity-dependent synaptic vesicle release at day 5 on rafts. Scale bar, 10 μm.

Advances in stem cell technology now allow generation of unprecedented models of human neurological disease states *in vitro*; however, the time and resources for such stem cell work presents a notable bottleneck. We reasoned that our micro-raft approach might allow screens using neurons derived from stem cells. We first generated neurons from H9 embryonic stem cells (ESC-neurons; Fig. 2A–E)^15^,^16^. We then cultured ESC-neurons onto raft arrays and released rafts within 2 days (see Fig. 1D). Neuronal marker expression and function (Fig. 2F–H) were confirmed by 5 days on released rafts, further validating that the raft micro-cultures remain functional after they are released from arrays.

### Mini-cultures versus well-plate cultures

**Figure 3:**
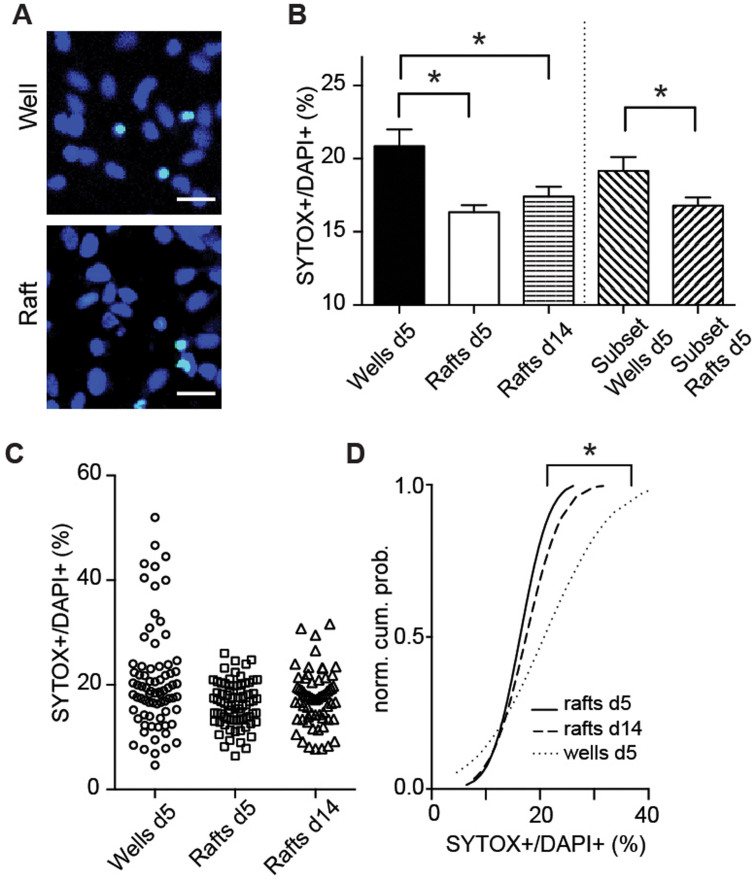
Human ESC-neuron mini-cultures on released micro-rafts are more viable and consistent compared with 384-well-plate cultures. (A) Example images of nuclei (DAPI, blue) and dead cells (SYTOX+, turquoise) from human ESC-neurons cultured for 5 days from the center of a well of a 384-well plate (top) and a released raft (bottom). Scale bar, 20 μm. (B) Graph showing the percentage of dead human ESC-neurons (SYTOX+/DAPI+) in wells of a 384-well-plate after 5 days in vitro (d5) and raft-mini-cultures at d5 and d14. This graph also shows a subset analysis of raft and well-plate cultures at d5 with equivalent cell densities. (C) Graph plot of all data points for wells and rafts shown in (*B*). (D) Normal cumulative distributions plots of rafts and wells presented in *(C)*. *p<0.05.

To evaluate the utility of raft micro-cultures for drug screening, we performed a viability assay using human ESC-neurons on released rafts compared with 384-well plates (Fig. 3). We assessed the neuronal viability of our ESC-neurons by determining the percentage of the dead cell marker SYTOX green^17^ –positive cells and using nuclear markers (DAPI or Hoechst) to label all cells (Fig. 3A). ESC-neurons were plated into a 384-well plate at 20,000 neurons/well (initial plating density: 1,800 neurons/mm^2^), as performed previously^18^. Within the wells of the well-plate, neurons are unevenly distributed, with the highest density around the periphery. We chose to compare neuron viabilities on day 5, the earliest time point we could detect neuronal function (Fig. 2H) from 300 μm x 300 μm regions around the center of each raft or well to simulate future automated imaging. By this day, there was a significantly lower fraction of dead cells on rafts than in wells (Fig. 3B). To ensure that the difference in viability was not due to the difference in neuron density at this time [612.5±32.5 live neurons/mm^2^ on raft vs. 291.4±21.9 live neurons/mm^2^ in wells; p<0.001], we compared the percentage of SYTOX+ neurons within a subset of rafts and wells containing equivalent densities of live neurons (see methods; Fig. 3B). Within these subsets, the difference in neuronal viability was retained (p < 0.05).

Next, we quantified sample-to-sample variability by plotting the percentages of SYTOX+ neurons of either rafts or wells on day 5 (Fig. 3C). Our analysis suggests that the percentage of dead cells is more consistent between rafts than between wells. Indeed, the cumulative distributions of percentages of SYTOX+ neurons were significantly different, with the distribution for the rafts significantly steeper, indicating less variability between rafts compared with wells (p<0.05). In addition, the between-sample consistency was maintained using rafts up to day 14 (Fig. 3C–D), whereas neurons in wells required daily media changes to survive (data not shown). This lack of maintenance requirements for raft arrays is a significant advantage over high-density well plates. These differences could be due to the higher amount of neuronal material required for well plating more quickly depleted the media of nutrients. Using rafts would drastically decrease the number of neurons per screen. Alternatively, over this time media composition in small volume well plates might be significantly altered by evaporation. Released rafts can be maintained in larger volume reservoirs of media less sensitive to evaporative effects and later moved to high content plates for imaging. Taken together, the increases in both neuronal viability and sample-to-sample reproducibility represent distinct advantages of raft arrays over conventional well-plate cultures.

### Measurement of synaptic phenotypes using human iPSC-neurons

**Figure 4:**
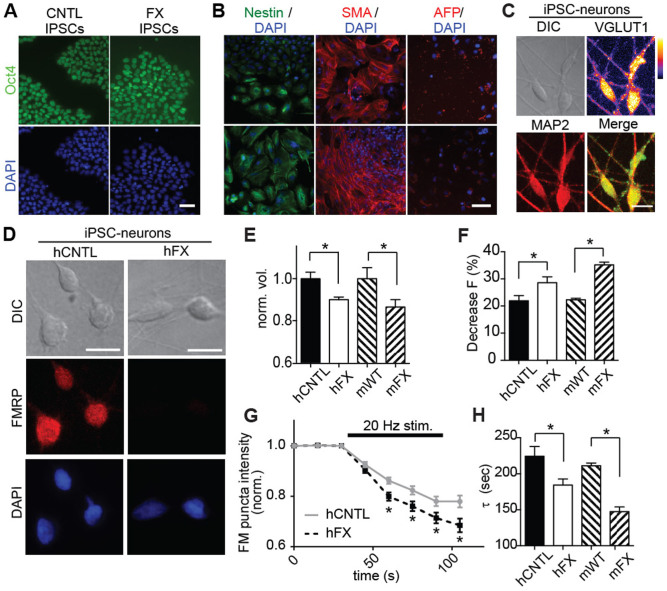
Raft mini-cultures can be used for cell-based assays of autism-linked neurodevelopmental disorders using both human iPSC-neurons and mouse model neurons. (A) Human iPSCs derived from fibroblasts of Fragile X or Control patients express the pluripotency marker Oct4 following reprogramming. (B) Both iPSC lines, differentiated as embryoid bodies for 3 weeks, expressed nestin, smooth muscle actin (SMA) or alpha fetoprotein, markers of ectoderm, mesoderm and endoderm, respectively. Scale bar, 50 μm. (C) Images of iPSC-neurons derived from FX individuals immunolabeled for excitatory neuronal markers VGLUT1 (‘fire’ look up table key shown; green in merged image) and MAP2 (red). Scale bar, 10 μm. (D) Images showing FMRP expression (red) of control and FX iPSC-neurons differentiated and grown on released rafts for 14 days (DAPI, blue). Scale bar, 10 μm. (E) Graph of the average FM4-64 puncta volumes for human FX iPSC-neurons (hFX; n = 477), control iPSC-neurons (hCNTL; n = 196), mouse FX hippocampal neurons (mFX; n = 208), and mouse wild-type hippocampal neurons (mWT; n = 165). (F) Graph of average percent unloading of FM5-95 puncta after electrical stimulation for hFX (n = 100), hCNTL (n = 90), mFX (n = 698), and mWT (n = 461). (G) Unloading curves of mean normalized fluorescence intensity of FM puncta over time (before and during stimulation) for hCNTL and hFX iPSC-neurons. (H) Bar graph of the mean fluorescence decay constant, τ, for FM puncta in response to electrical stimulation for both human and mouse Fragile X cellular models and controls; samples size are described in *(F)*. *p<0.05.

While sample-to-sample consistency between screens is vital to drug discovery, it is also critical that the model system recapitulates human disease phenotypes^9^. Often this is accomplished by harvesting neurons from readily-available transgenic rodent lines, but human iPSCs are increasingly used to generate neurons from neurological patients. To confirm that raft mini-cultures could successfully be used as a platform to model human disease phenotypes, we used iPSC-neurons from a patient with Fragile X (FX iPSC-neurons), the most prevalent inherited form of intellectual disability^19^, and we functionally compared these neurons to iPSC-neurons from a neurotypical individual (CNTL iPSC-neurons) (Fig. 4A–H). We concentrated on FX for these proof-of-principle experiments because: 1) the genes underlying FX are well-known, 2) the disease state can be confirmed by the lack of Fragile X mental retardation protein (FMRP) expression, and 3) there is a well-characterized, transgenic rodent model (the FMR1 KO mouse) for comparison. Importantly, iPSC-neurons exhibited neuronal markers (Fig. 4C), and neither the reprogramming nor differentiation altered the level of FMRP expression (Fig. 4D).

We plated FX and CNTL iPSC-neurons onto raft arrays, released them after 1 day, and then used lipophilic FM dyes to evaluate differences in presynaptic function at 14 days post-plating^20^,^21^. We found a significant decrease in the volume of FM puncta following activity-dependent loading in FX iPSC-neurons, compared to CNTL-iPSC neurons (Fig. 4E), suggesting a smaller recycling pool or a reduction in the rate of endocytosis. In addition, we found significantly enhanced unloading kinetics in FX iPSC-neurons, compared to CNTL iPSC-neurons (Fig. 4F–H). Importantly, these differences were also found between primary neurons from FMR1 KO mice and WT mice (Fig. 4E–H), and are consistent with the previous findings that FX neurons have decreased numbers of synaptic vesicles^22^ and a higher probability of neurotransmitter release^23^. These results demonstrate that, despite the reduced number of neurons in micro-rafts compared to culture-wells, there remains sufficient statistical power to screen and identify reproducible functional phenotypes.

### Drug screening using released micro-rafts

**Figure 5:**
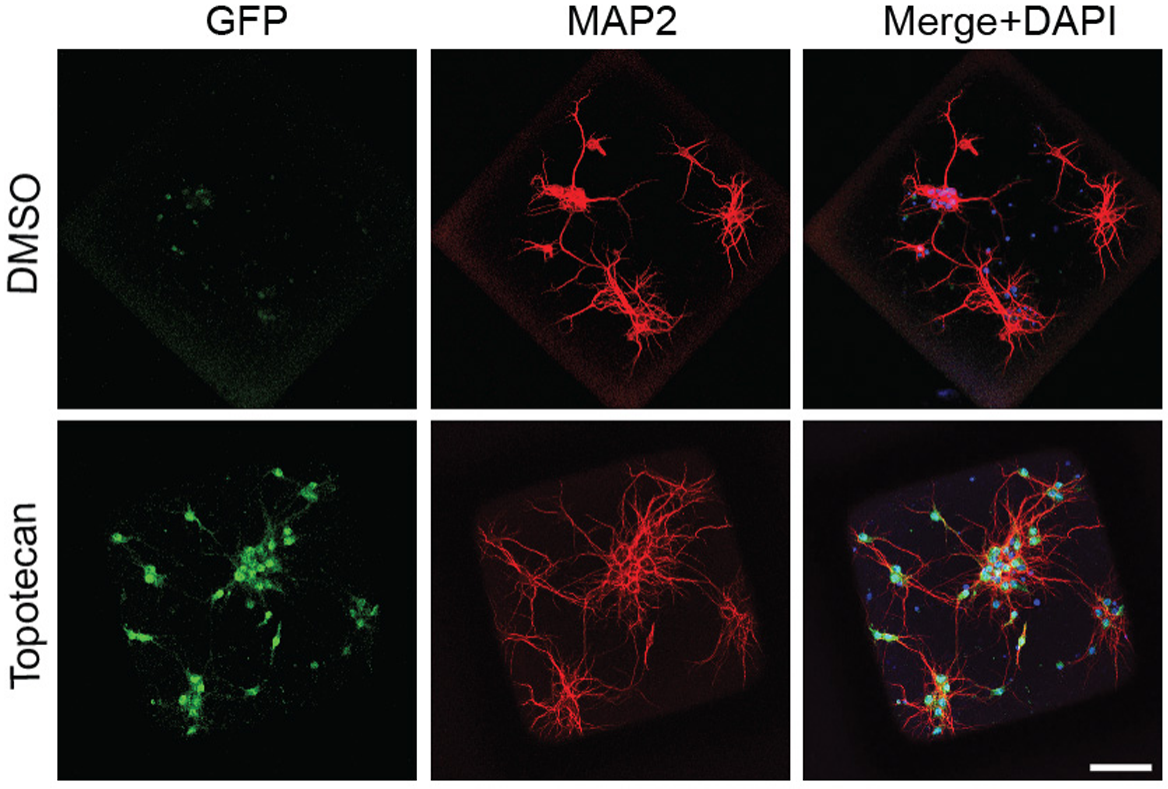
Topotecan induced the unsilencing of Ube3a-YFP (immunolabeled using an anti-GFP antibody, green) expressed on the normally silent paternal allele in mouse cortical neurons mini-cultures grown on rafts. 300 nM Topotecan, 72 hours. Scale bar, 100 μm.

Finally, we performed a proof-of-concept study to demonstrate the use of raft arrays in a drug screening assay, by reproducing previous results from a much lower throughput screen^18^. We used embryonic neurons from a mouse model containing a Ube3a-YFP transgene within the normally silent paternal allele previously used to screen potential therapeutic compounds for Angelman syndrome^18^. Just as in the previous screen, the topoisomerase inhibitor, topotecan, successfully increased expression of YFP in raft mini-cultures (Fig. 5), indicating that topotecan activates the normally dormant paternal allele of Ube3a. Together, these data show that drug treatment in raft mini-cultures reproduces findings reported using traditional well-plate cultures.

## Discussion

Micro-raft arrays allow significantly enhanced sample sizes and a reduction in the number of neurons needed from existing supply-limited sources. An extraordinary amount of time and resources are dedicated to generating neuron cultures. The lack of efficient differentiation protocols for stem cells and the length of time required to differentiate and maintain these neurons (>30 days), present bottlenecks for their use in high-throughput screening^9^. For neurons obtained directly from embryonic mice, which yield the largest number of live cortical neurons, the generation of animals presents a major bottleneck, compounded by poor reproductive success (<50%), small litter size, long gestation times, and short fertile windows. For example, cortical neurons harvested from a single mouse embryo (~3,000,000 neurons) could generate 4,800 rafts; the same number of samples using 384-well-plates would require 32 embryos and multiple litters.

Micro-raft arrays also overcome key technical challenges in high-throughput screening using neurons. First, they overcome difficulties with evaporation that plague traditional long-term micro-cultures in high-density well plates^9^. After micro-rafts are released, they can be cultured for extended lengths of time in large media volumes that are not affected significantly by evaporation until they are ready to be transferred for screening and imaging. Second, the use of arrays reduces the plating time, supplies, and reagents, because dissociated neurons are plated *en masse* onto each disposable array (~3 arrays per embryonic mouse cortex) (movie S1).

Finally, additional experimental possibilities result from advantages in handling capabilities. These arrays are produced rapidly and reproducibly^11^, do not require large investment in expensive equipment, and are compatible with existing high-throughput fluid handling equipment, established compound libraries, and high-resolution fluorescence microscopy. The consistent boundaries of the raft will facilitate automated detection for high-throughput imaging. Thus, the raft arrays are well-suited for both basic science experimentation and high-throughput screens. In summary, these data show that micro-raft arrays can dramatically increase the scale of screening studies in neurons and open up neuron-based large-scale screening to the broad research community.

## Methods

### Stem Cells

The NIH-approved, human embryonic stem cell (ESC) line H9 (WA09) was obtained from WiCell Research Institute (Madison, WI). H9 ESCs were maintained as undifferentiated colonies on growth factor reduced Matrigel (BD Biosciences) in mTeSR1 media (StemCell Technologies). Media was changed daily. Cells were passaged every three days with 0.5 mM EDTA (340 mOs).

Human iPSCs were generated from purchased human fibroblast lines. The Fragile X positive fibroblast line, GM05131A was purchased from Coriell Institute for Medical Research at passage 11 and non-Fragile X line CCD-1079sk was obtained from ATCC and used for experiments at passage 7. Both lines were maintained and cultured according to the vendor's instructions. To induce pluripotency, both fibroblast lines were plated into 6-well plate (200,000 cells/well) and maintained overnight in DMEM (Gibco) supplemented with 10% FBS (Gibco). Cells were then infected with lentiviral particles carrying the following reprogramming genes: Oct4, Sox2, Klf4, cMyc, Lin28 and Nanog. Individual hiPS clones were selected after 14 to 21 days, expanded and characterized using the germ layer characterization kit (Millipore). To verify their pluripotency hiPS clones were plated onto 10 cm tissue culture dishes and grown to 80% confluency. At this point cells were dissociated using EDTA and grown for three weeks as embryoid bodies (EBs) in suspension on Petri dishes in IMDM (Lonza) supplemented with 10% FBS (HyClone). In addition, Rock inhibitor (Selleck Chemicals) was maintained in the medium for the first 24 hours. After three weeks embryoid bodies were seeded on Matrigel-coated 6-well plates, fixed and processed for expression of germ layer biomarkers (Fig. 2).

### Differentiation and maintenance of human ESC- and iPSC-neurons

We adapted a previously published protocol for physiologically-active, glutamatergic neurons of the forebrain^16^. Stem cells were maintained and expanded in mTeSR1 for 1 week (passaged on day 3 and 6) then dissociated to single cells using 0.5 mM EDTA (340 mOs) and plated in suspension on Petri dishes in DMEM/F12 plus 20% knockout serum replacement, 1 mM L-glutamine, 1% nonessential aminoacids and 0.1 mM beta-mercaptoethanol (EB media). EB media was half-changed daily. Between days 1 and 5 of differentiation, media was supplemented with: SB431542 (10 μM), LDN193189 (100 nM), XAV939 (10 μM) and retinoid acid (10 μM). On day 5 of differentiation the EBs (30-50/well) were seeded on 6-well Matrigel-coated plates in N2B27 medium, consisting of a 1:1 mixture of N2 medium (DMEM/F12 GlutaMAX, 1 X N2, 5 ug/ml insulin, 1 mM L-glutamine, 100 μM nonessential aminoacids and 100 μM beta-mercaptoethanol) and B27-containing media (Neurobasal medium, 1 X B27 and 200 mM L-glutamine supplemented with SB431542 (10 μM), LDN193189 (100 nM) and XAV939 (10 μM) and 10 μM Retinoic acid. On day 7 the medium was changed to N2B27 supplemented with 10 μM Retinoic acid. From day 7 until day 24 the medium (N2B27-RA) was replaced daily.

At day 24, the neuroepithelial cells in the center of colonies that had formed neural tube-like rosettes and were attached loosely were manually lifted, dissociated either manually or with Accutase (Life Technologies) and then seeded on poly-L ornithine coated micro-raft arrays or well plates in N2B27 medium overnight. The following day, micro-rafts were released from the array and the medium was changed to N2B27 supplemented with BDNF (10 ng/ml) for all cells. For ease of understanding, days were renumbered such that day 1 corresponded to the first day on raft arrays (Fig. 2A) and day numbers in throughout the text use this numbering system unless otherwise stated. Stem cell neurons on both released rafts and in wells began extending neurites by day 2-3. Stem cell neurons were then maintained for up to 14 days post plating as described.

### Stem cell-neuron viability assay

To assess viability of H9-derived neurons, cells were plated on either micro-raft arrays (1 million neurons/array) or in wells of a 384-well plate (20,000 neurons/well) in N2B27 containing BDNF. Rafts were released and transferred on day 2 as described. On either day 5 or day 14, cells were treated with SYTOX green (1 μM; Life Technologies) and either NucBlue (2–3 drops/mL; Molecular Probes) or DAPI (300 nM; Life Technologies) for 5–10 minutes to visualize dead/dying cells and all cells, respectively. Neurons were then rinsed with PBS and mounted with Fluoromount-G (Southern Biotech) for imaging and assessment. Live neuron densities were extrapolated from the number of SYTOX-negative neurons within a 300 μM x 300 μM image from the center of the raft or well. To standardize neuronal density when calculating neuronal viability, percentages of SYTOX-positive neurons were also compared from a subset of rafts or wells containing between 100 and 300 live neurons per 300 μM x 300 μM image.

### Mouse cultures

All experimental procedures were carried out in accordance with the University of North Carolina at Chapel Hill Institutional Animal Care and Use Committee (IACUC). Hippocampi were dissected from both postnatal day 1-3 WT- (C57BL/6; Jackson Labs) and FMR1 knockout mice (on the C57BL/6 background; Jackson Labs) in ice cold dissociation media (DM; containing NA2SO4 [8.2 mM], K2SO4 [3 mM], MgCl2 [0.58 mM], CaCL2 [25.2 nM], HEPES [0.1 mM], Glucose [0.2 mM], and phenol red [0.001%]). Hippocampi were then incubated (15 minutes at 37°C) 2 times in 5 mL filtered Cystine-activated Papain solution (CAPS; 10 mL DM plus: L-cystine [Sigma; 3.2 mg] and Papain [Sigma; 17units/mL]; pH 7.4). The CAPS was replaced with DM containing 10% fetal bovine serum to inactivate papain. Hippocampi were then washed with DM and then with Neurobasal media (NBM; Neurobasal A media [Gibco] plus: B27 [50x; Gibco; 2%], GlutaMax [100x; Gibco, 1%], and Anti-anti [100x; Gibco, 1%]). Next, hippocampi were manually dissociated in fresh NBM, centrifuged (67 xg) for 7 minutes at 4°C, and resuspended (12 million neurons/mL) in NBM. Neurons were then plated on poly-D-lysine-coated raft arrays (1 million neurons/array). Primary cortical neuron cultures from *Ube3a-YFP* knock-in mice were prepared as previously described^18^. Rafts were then released the following day (DIV 2) and maintained in NBM until DIV14 as described.

### FM dye assays

To assess puncta-unloading rates, released rafts with either stem cell neurons or mouse neurons were transferred via pipette to a devised micro-chamber (8 mm diameter polydimethylsiloxane; PDMS; reservoir on a glass coverslip). Cell culture media was replaced with HEPES-buffered solution (HBS; 119 mM NaCl, 5 mM KCl, 2 mM CaCl_2_, 2 mM MgCl_2_, 30 mM glucose, 10 mM HEPES) and allowed to recover for at least 30 min at 37°C. HBS was replaced with the FM dye loading solution containing 10 μM *N*-(3-trimethylammoniumpropyl)-4-(6-(4-(diethylamino)phenyl)hexatrienyl)pyridinium dibromide (FM 5-95; Invitrogen), 20 μM CNQX (Tocris Bioscience), 50 μM d-AP5 (Tocris Bioscience), and high KCl buffered saline (90 mM KCl, 34 mM NaCl, 2 mM CaCl_2_, 2 mM MgCl_2_, 30 mM glucose, 10 mM HEPES) for 1 min to allow for endocytosis of the dye. Loading solution was then removed and neurons were rinsed with 10 μM FM 5-95 in HBS for 1 min. Next, neurons were rinsed three times with a high-Mg^2+^, low-Ca^2+^ solution (106 mM NaCl, 5 mM KCl, 0.5 mM CaCl_2_, 10 mM MgCl_2_, 30 mM glucose, 10 mM HEPES) containing 1 mM Advasep-7 (Biotium) for 1 min. Finally neurons were rinsed three times with HBS containing 20 μM CNQX and 50 μM d-AP5 for 1 min. Unloading was performed using electrical field stimulation. Positive and negative electrodes were placed in on either side of the raft. Stimulation was generated with a two-channel stimulus generator (STG4002; AD Instruments) in current mode with an asymmetric waveform (−480 μA for 1 ms and + 1600 μA for 0.3 ms) at 20 Hz for 600 pulses. *Z*-stacks (>30 slices) were captured every 15 s using auto-focus for each time-point to prevent drifting. At least five baseline images were acquired before starting electrical stimulation.

To assess puncta volumes, neurons were loaded as described above with the fixable version of FM 4–64 (Invitrogen) replacing FM 5–95. Following rinses, neurons were fixed with 4% paraformaldehyde in phosphate buffered saline (PBS) containing 40 mg/ml sucrose, 1 μm MgCl_2_, and 0.1 μm CaCl_2_ for 15 minutes, rinsed 3 times with PBS, then mounted in Fluoromount-G for imaging.

### Immunocytochemistry

Neurons on rafts or in well plates were fixed with 4% paraformaldehyde in PBS containing 40 mg/ml sucrose, 1 μm MgCl_2_, and 0.1 μm CaCl_2_ for 20–45 min. Neurons were permeabilized in 0.25% Triton X-100 for 15 min then blocked in PBS containing 10% goat serum for 15 min. Primary antibodies to β-tubulin (1:2000; chicken; Aves Labs), MAP2 (1:1000; rabbit; Millipore), VGLUT1 (1:200; mouse; NeuroMab), and FMRP (1:10; mouse; 2F5-1 [developed by Tartakoff, A.M./Fallon, J.R. and obtained from the Developmental Studies Hybridoma Bank developed under the auspices of the NICHD and maintained by The University of Iowa, Department of Biology, Iowa City, IA 52242]) were diluted in PBS with 1% goat serum and incubated for 1 h at room temperature. AlexaFluor goat anti-mouse, anti-rabbit, and anti-chicken antibodies conjugated to fluorophores with 488 nm, 568 nm, or 633 nm excitation wavelengths (1:1000; Invitrogen) were diluted in PBS and incubated for 1 h at room temperature. Immunostaining for neurons from *Ube3a-YFP* knock-in mice were performed as previously described^18^.

### Microscopy

Images were acquired using a spinning disk confocal imaging system (CSU-X1, Yokogawa) configured for an Olympus IX81 zero-drift microscope (Revolution XD, Andor Technology). Light excitation was provided by 50 mW, 488 nm; 50 mW, 561 nm; and 100 mW, 640 nm lasers. The following bandpass emission filters (BrightLine, Semrock) were used: 525/30 nm (TR-F525-030), 607/36 nm (TR-F607-036), 685/40 nm (TR-F685-040). For FM dye imaging, we used a spinning disk confocal imaging system with excitation at 561 nm (25% intensity) and with the 685/40 nm emission filter. We used 2×2 binning to reduce the laser intensity and acquisition time for each frame. Each *z*-stack was obtained in ~5 s.

### Image processing and analysis

Confocal slices were sum projected in ImageJ and converted to 8-bit depth. For the FM image analysis, we thresholded the first frame of the *z*-stack to a minimum pixel value of 30. We analyzed puncta >2 pixels^2^ and measured the intensity of each of these puncta throughout the registered stack. The intensity of each puncta was normalized to the frame preceding stimulation and slope normalized to baseline. Puncta that unloaded >5% after 1 min were classified as “unloading” and were included in the analysis. For puncta volume measurements, z-stacks were analyzed using ImageJ plug-in “Foci Picker3D”^24^ using the following settings: uniform background 5, tolerance setting 5, and minimum pixels in focus 20.

### Statistics

Statistical analyses were performed using GraphPad Prism. When comparing distributions we used the non-parametric t-test (Kolmogorov-Smirnov test). Two-tailed unpaired Student's *t* test was used when comparing means for two conditions. Error bars represented the standard error of the mean (SEM) and the threshold for statistical significance was set at p<0.05 throughout the study.

## Author Contributions

M.N. designed and performed experiments and wrote the manuscript. R.D. designed and performed experiments. A.M.M. designed and performed experiments. Y.W. designed experiments. B.D.P. designed experiments and helped write the manuscript. N.L.A. designed experiments. A.M.T. designed experiments and wrote the manuscript.
